# Malaria in a 2-Month-Old HIV-Exposed Nigerian Infant: Challenges of Care

**DOI:** 10.1177/2325958219849052

**Published:** 2019-05-23

**Authors:** Olusola Adetunji Oyedeji

**Affiliations:** 1 Ladoke Akintola University of Technology Teaching Hospital, Osogbo, Osun, Nigeria

**Keywords:** malaria, HIV, exposure, infancy

## Abstract

**Background:**

Reports on malaria and HIV coinfections in exposed infants from tropical countries are scarce.

**Results:**

The case of a 2-month-old HIV-exposed Nigerian infant who presented with intermittent fever at a Nigerian tertiary hospital is reported. The rarity of the case and the challenges associated with making the diagnosis informed our decision to report the case.

**Conclusion:**

Diagnosing malaria in HIV-exposed infants in early infancy requires a high index of suspicion, good knowledge of the clinical presentation, and appropriate microbiological investigations for sepsis and malaria. Further studies need to be conducted on the association between malaria and HIV exposure.

**What Do We Already Known About This Topic?**

Malaria is rare in children aged below 3 months and considered an unlikely infection.

**How Does Your Research Contribute to this Field?**

Malaria should be considered as a possible cause of fever in HIV-exposed infants aged <3 months.

**What Are Your Research Implications toward Theory, Practice, or Policy?**

Screening for malaria should be routine in HIV-exposed febrile children aged <3 months.

## Introduction

Malaria is a common cause of morbidity and mortality in Nigerian children.^1,2^ It is however rare in infants aged less than 6 months in malaria endemic settings. We recorded this case of malaria in *an HIV*-*exposed 2-month-old infant.* The reasons adduced for the rarity of malaria in infants aged less than 3 months include the protective placental barrier and passive transfer of antimalaria antibodies from the mother to fetal circulation across the placenta in semi-immune mothers.^3,4^ Another factor hindering the survival of malaria parasites in breastfed infants include the deficiency of para-amino benzoic acid in breast milk, which is needed for malaria parasite nucleic acid metabolism. *Fetal hemoglobin also* inhibits growth and survival of malaria parasites.^3^ The current Nigerian guidelines for management of HIV and previous research studies on this subject suggest that malaria is rare in HIV-infected or exposed infants aged below 6 months.^4-7^ The purpose of this report is to discuss the clinical presentation of malaria in an HIV-exposed infant and provide information that may aid diagnostic acumen and proper case management. This report is also written with a view to canvass for better planned studies on the association between malaria and HIV exposure or infection in infants.

## Case Report

A 2-month-old HIV-exposed male Nigerian infant presented at the pediatric antiretroviral clinic in the company of his mother for follow-up. His mother complained of fever of a week duration occurring every 2 to 3 days, with the child appearing unwell when febrile and well when afebrile. An episode of nonprojectile vomit containing recently ingested milk formula was associated with this fever. There was no history of irritability, excessive crying, jaundice, reduced feeding, *or history suggestive of dysuria in the patient.* Neither was there a cough, rhinorrhea, or dyspnoea. There was no history suggestive of prolonged rupture of membrane, chorioamnionitis, or maternal infections around the delivery period. *The infant had never been transfused with blood or blood products.* The *mother of the patient was a 38 -year-old nursing officer* who was diagnosed to be HIV infected 16 years previously. Mum had adhered to her medications since highly active antiretroviral therapy (HAART) was initiated 7 years prior to the admission of the baby. The father, a 50-year-old accountant, was also HIV infected and had also initiated HAART. The family setting is monogamous and the child is the second in a family of 3.

The index patient was delivered at term by elective cesarean section with a birth weight of 2.8 kg and there was no history suggestive of birth asphyxia. The infant was initially breastfed for 2 weeks and thereafter fed with infant formula because the mother could not bear her neck pains due to cervical spondylosis. Nevirapine prophylaxis was administered from birth for the first 6 weeks of life. Co-trimoxazole prophylaxis was then commenced at the age of 6 weeks.

General examination at presentation revealed an afebrile male infant with a temperature of 37.5°C and a weight of 3.6 kg. He was pale but not jaundiced or cyanosed. He was well hydrated and systemic examination revealed a heart rate of 144/minute with normal heart sounds. Both hepatic and splenic enlargement of 4 cm each were detected on abdominal examination. No abnormalities were detected on examination of the respiratory or central nervous systems. A diagnosis of sepsis was entertained and the patient was admitted and treated with intravenous cefuroxime before complete blood count investigation. Fever resolved 2 days after commencing antibiotics and the patient was discharged home on oral cefuroxime. Complete blood count investigation conducted at the age of 2 months revealed a packed cell volume of 29% and total white cell count of 9800 mm^3^. The differential lymphocyte and neutrophils counts were 84% and 16%, respectively. Blood film for malaria parasite screening was not done because malaria was not entertained. Blood investigations for HIV by polymerase chain reaction were negative.

The mother re-presented a week later with complaints of fever recurring every 2 to 3 days. Examination findings were similar to those observed before discharge. The unresolved fever pattern was not reported on time by the mother because she felt that the fever would abate with cefuroxime administration. A diagnosis of malaria was therefore entertained, which was confirmed after detection of trophozoites of *Plasmodium falciparum* in the peripheral blood film following microscopy. The infant was readmitted and treated with oral quinine. A remarkable improvement in his condition was thereafter noted with the intermittent fever resolving by crisis within 24 hours of administration of quinine. The packed cell volume of 26% at readmission increased to 32% on the fourth day of quinine therapy. The child remained well and was discharged home 2 days after.

## Discussion

The diagnosis of malaria in a 2-month-old infant of an HIV-infected mother resident in a malaria endemic setting is unusual. The HIV infection in the mothers could have probably predisposed this infant to congenital malaria. Studies examining the association between malaria and HIV exposure in children are scant, but a previous study has shown that maternal HIV infection was associated with increases in the cord blood *P falciparum* infection and decreased maternal antimalaria specific antibodies.^8^ Early cessation of breast feeding and intake of infant formula at the age of 2 weeks could have further contributed to the predisposition of the index case to malaria. Breast milk is deficient in para-amino benzoic acid which is needed to inhibit the thriving malaria parasites.^3^ The present case also shows that the antimalarial efficacy of cotrimoxazole is not 100%,^9,10^ due to the fact that tertian or quartan fever persisted from the sixth to the eighth week of life of this infant despite adherence to the daily cotrimoxazole prophylaxis from the sixth week of life.

The onset of tertian or quartan fever in a malaria endemic region is highly suggestive of *P falciparum* or malariae infection. Detection of *P falciparum* parasites in the peripheral blood film confirmed malaria infection in the case reported. Clinical recovery to quinine monotherapy further supports the diagnosis of malaria. Sepsis is a common differential for febrile infants at the age of 2 months and it was considered as the most likely cause of fever in this infant, thus necessitating initial use of antibiotics only. Undue fixation of sepsis as the only differential of fever in infants may lead to a delay or missed diagnosis of malaria. This underscores the importance of a good clinical acumen and high index of suspicion.

Confirming or ruling out sepsis can be a herculean task in resource restricted settings where diagnostics can be quite expensive and this may lead to being selective in carrying out tests indicated in screening for sepsis. Complete blood counts and blood film for malaria examination costs approximately US$5, while blood cultures costs as much as US$15 in our setting. Thus, selective and sequential running of tests is often the practice for the purpose of cost-effectiveness, but the disadvantage lies in the consequences of delayed or missed diagnosis of diseases. The lesson of this report is to extend the pragmatic practice of examining blood films for malaria parasites in infants presenting with fever in malaria endemic communities to HIV-exposed newborns. Additional studies are required to explore the association between malaria and HIV in exposed infants.
